# Hesperetin Inhibits Expression of Virulence Factors and Growth of *Helicobacter pylori*

**DOI:** 10.3390/ijms221810035

**Published:** 2021-09-17

**Authors:** Hyun Woo Kim, Hyun Jun Woo, Ji Yeong Yang, Jong-Bae Kim, Sa-Hyun Kim

**Affiliations:** 1 Department of Biomedical Laboratory Science, College of Health Sciences, Yonsei University, Wonju 26493, Korea; amaranth1001@nate.com (H.W.K.); kimjb70@yonsei.ac.kr (J.-B.K.); 2 Department of Clinical Laboratory Science, Semyung University, Jecheon 27136, Korea; taesube@nate.com; 3 Division of Crop Foundation, National Institute of Crop Science (NICS), Rural Development Administration (RDA), Wanju 55365, Korea; yjy90@korea.kr

**Keywords:** hesperetin, *Helicobacter pylori*, flavanone, natural compound

## Abstract

*Helicobacter pylori* (*H. pylori*) is a bacterium known to infect the human stomach. It can cause various gastrointestinal diseases including gastritis and gastric cancer. Hesperetin is a major flavanone component contained in citrus fruits. It has been reported to possess antibacterial, antioxidant, and anticancer effects. However, the antibacterial mechanism of hesperetin against *H. pylori* has not been reported yet. Therefore, the objective of this study was to determine the inhibitory effects of hesperetin on *H. pylori* growth and its inhibitory mechanisms. The results of this study showed that hesperetin inhibits the growth of *H. pylori* reference strains and clinical isolates. Hesperetin inhibits the expression of genes in replication (*dna*E, *dna*N, *dna*Q, and *hol*B) and transcription (*rpo*A, *rpo*B, *rpo*D, and *rpo*N) machineries of *H. pylori*. Hesperetin also inhibits the expression of genes related to *H. pylori* motility (*flh*A, *fla*A, and *flg*E) and adhesion (*sab*A, *alp*A, *alp*B, *hpa*A, and *hop*Z). It also inhibits the expression of urease. Hespereti n downregulates major virulence factors such as cytotoxin-associated antigen A (CagA) and vacuolating cytotoxin A (VacA) and decreases the translocation of CagA and VacA proteins into gastric adenocarcinoma (AGS) cells. These results might be due to decreased expression of the type IV secretion system (T4SS) and type V secretion system (T5SS) involved in translocation of CagA and VacA, respectively. The results of this study indicate that hesperetin has antibacterial effects against *H. pylori*. Thus, hesperetin might be an effective natural product for the eradication of *H. pylori.*

## 1. Introduction

*Helicobacter pylori* (*H. pylori*) is a Gram-negative, curved bacterium. It is well known as an important human pathogen. *H. pylori* causes various gastrointestinal diseases such as gastritis, chronic gastritis, peptic ulcer, mucosa-associated lymphoid tissue (MALT) lymphoma, and gastric adenocarcinoma [1]. The prevalence of *H. pylori* varies widely depending on age, ethnicity, socioeconomic status, dietary habits such as food, alcohol consumption, and smoking status [2]. In particular, East Asian countries are at high risk of gastric cancer due to the high rates of *H. pylori* infection [3]. Therefore, a concerted effort for the eradication of *H. pylori* infection is necessary for health promotion worldwide.

DNA replication and transcription are vitally important processes for the survival and propagation of all living organisms including *H. pylori*. DnaA and DnaB form the complex of DNA-unwinding helicase [4,5]. DnaG primase can synthesize primers [6]. DNA polymerase III consists of core polymerases (DnaE, DnaQ, and HolE), sliding clamp (DnaN), and multiprotein clamp-loader (DnaX, HolA, HolB, HolC, and HolD), all of which are necessary for an organism to function properly [7,8]. Bacterial RNA polymerase consists of four subunits: α, β, β’, and ω subunits (α2ββ’ω) [9]. The *rpo*A and *rpo*B genes encode α and β subunits, respectively. Both *rpo*N and *rpo*D genes encode the σ factor.

The motility of *H. pylori* plays an important role in the movement of *H. pylori* toward the gastric epithelium for successful colonization [10,11]. *H. pylori* has lophotrichous flagella. FlaA and FlaB are flagellin proteins that comprise the filament structure of flagella [12,13]. FlgE is a hook protein that connects the flagellar filament to the export domain of the basal body [14]. FlhA is involved in the expression regulation and export of flagellin proteins [15].

The adhesion of *H. pylori* is an important step in establishing a successful infection by protecting *H. pylori* from clearance mechanisms such as gastric peristalsis and mucus flow. BabA (HopS) is a major adhesin and SabA (HopP) is the second most known adhesin of *H. pylori* [16,17]. OipA (HopH) promotes IL-8 secretion and causes inflammation [18]. AlpA (HopC) and AlpB (HopB) are adherence-associated lipoproteins and HpaA is a surface-located lipoprotein [19,20,21,22]. HopZ is involved in colonization [23,24].

One of the most studied virulence factors of *H. pylori* is cytotoxin-associated gene A (CagA). After it is injected into host cells, it can affect cell shape and proliferation [25]. CagA is injected directly into host cells through type IV secretion system (T4SS) encoded in *cag*PAI [26]. The T4SS consists of 12 subunits, including 11 VirB proteins (VirB1–VirB11) and VirD4 protein [27,28,29].

Vacuolating cytotoxin A (VacA) is another well-studied virulence factor of *H. pylori.* It is a toxin secreted by type V_a_ secretion system (T5_a_SS). It is known to induce cytoplasmic vacuole formation [30]. The secretion system subunit protein A (SecA) is an especially important regulatory protein because it is an ATPase that provides energy for the translocation of VacA to the cell membrane [31].

**Figure 1:**
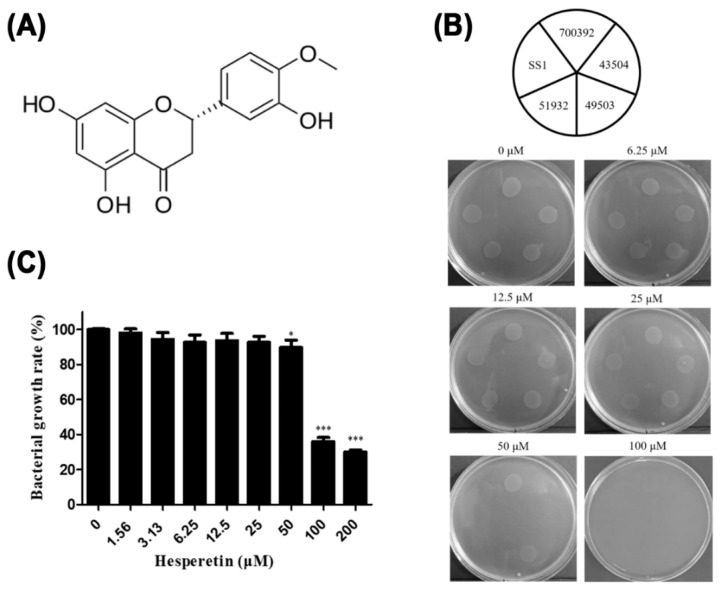
Antibacterial activity of hesperetin against *H. pylori*. (**A**) Chemical structure of hesperetin. (**B**) Minimal inhibitory concentrations of hesperetin against five *H. pylori* reference strains (ATCC 49503, ATCC 43504, ATCC 51932, ATCC 700392, and SS1) were determined by the agar dilution method. (**C**) The minimal inhibitory concentration of hesperetin against *H. pylori* ATCC 49503 strain was confirmed by the broth dilution method. Data are presented as mean ± SEM. Results from triplicate experiments were analyzed by Student’s *t*-test (* *p* < 0.05 and *** *p* < 0.001).

Hesperetin is a natural flavanone. An aglycone of hesperidin has been found in citrus fruits such as lemon, lime, and orange. The chemical structure of hesperetin is shown in Figure 1A. Hesperetin has been reported to show various beneficial effects, including antioxidant, anti-inflammatory, and chemopreventive effects and anti-carcinogenic properties [32,33]. There are also several studies that have reported the antibacterial effect of hesperetin against various bacteria including *Escherichia coli*, *Salmonella enterica*, and *Pseudomonas putida* [34,35]. Lee et al. [36] have reported that plant flavonoids including hesperetin possess inhibitory effects on *H. pylori*. However, the mechanism by which hesperetin inhibits *H. pylori* has not been elucidated yet. There are many studies reporting on flavonoids and their anti-virulence potential against *H. pylori* [37,38,39]. Therefore, the objective of this study was to determine the inhibitory effect of hesperetin on *H. pylori* growth and its inhibitory mechanism. The results of this study suggest that hesperetin is an effective natural product for the eradication of *H. pylori*.

## 2. Results

### 2.1. Inhibitory Effect of Hesperetin on the Growth of H. pylori by Downregulating Replication and Transcription Genes

The agar dilution test was performed to determine the minimum inhibitory concentration (MIC) of hesperetin against *H. pylori*. Mueller–Hinton agar plates containing 10% bovine serum and various concentrations (0, 6.25, 12.5, 25, 50 and 100 μM) of hesperetin were prepared. Five *H. pylori* reference strains (ATCC 49503, ATCC 43504, ATCC 700392, ATCC 51932, and SS1) were grown on agar plates for 72 h. According to the agar dilution test, MICs of hesperetin against ATCC 43504, ATCC 51932, and SS1 strains were 50 μM while those against ATCC 49503 and ATCC 700392 strains were 100 μM (Figure 1B). To confirm that hesperetin could inhibit the growth of *H. pylori* clinical isolates as well as reference strains, 46 clinical isolates of *H. pylori* were collected from gastric biopsies and MICs of hesperetin against the isolates were determined with the agar dilution test. Results showed that the MIC of hesperetin was 6.25 μM against 8.7% (4/46), 12.5 μM against 6.5% (3/46), 25 μM against 6.5% (3/46), 50 μM against 69.6% (32/46), and 100 μM against 8.7% (4/46) of these clinical isolates (Table 1). Because *H. pylori* strains were grown in the broth for subsequent experiments, the MIC of hesperetin was also determined by the broth dilution test. Among five *H. pylori* reference strains, ATCC 49503, which is known as the most virulent CagA(+)/VacA(+) strain, was used in these experiments. As a result of the broth dilution test, the growth of *H. pylori* was significantly inhibited by hesperetin at a concentration above 100 μM (Figure 1C). These results confirm that hesperetin has an antibacterial effect on *H. pylori*. Particularly, these results showed that hesperetin not only possesses antibacterial effects on *H. pylori* strains but also exerts reference-similar antibacterial effects on the clinical isolates of *H. pylori*.

**Figure 2:**
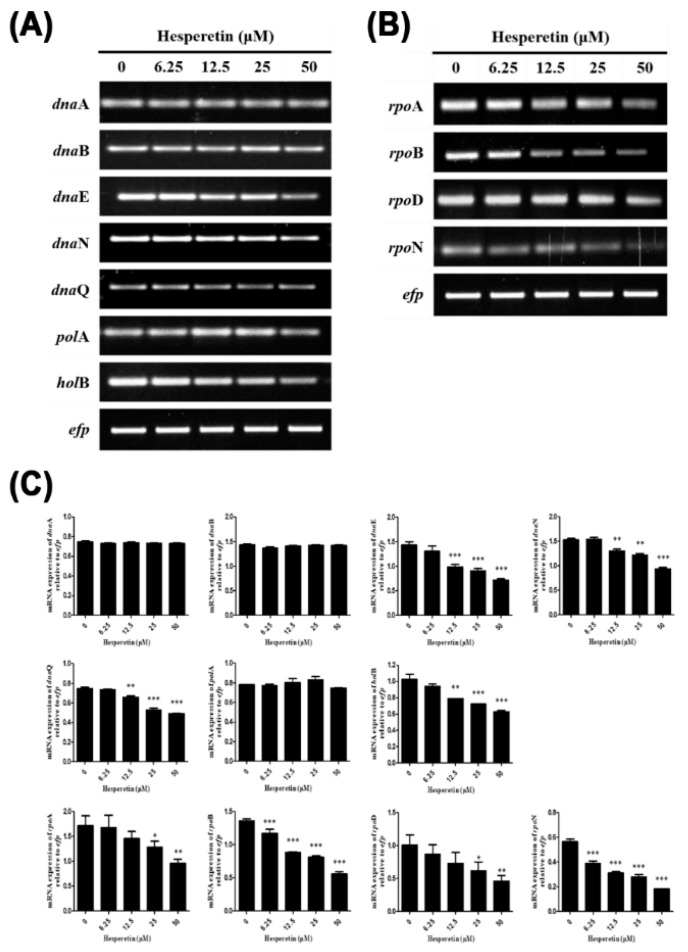
Downregulation of replication and transcription genes of *H. pylori*. RNA was subjected to RT-PCR to detect mRNA expression levels of genes of (**A**) the replication machinery (*dna*A, *dna*B, *dna*E, *dna*N, *dna*Q, *pol*A, and *hol*B) and (**B**) the transcription machinery (*rpo*A, *rpo*B, *rpo*D, and *rpo*N). The expression of *efp* was used as an internal control. (**C**) Each band intensity was normalized to *efp*. Data are presented as mean ± SEM of triplicate experiments and were analyzed by Student’s *t*-test (* *p* < 0.05, ** *p* < 0.01, and *** *p* < 0.001).

The expression levels of genes in replication and transcription machineries of *H. pylori* were evaluated to determine the mechanism by which hesperetin could inhibit the growth of *H. pylori*. *H. pylori* was treated with a sub-MIC concentration of hesperetin (0, 6.25, 12.5, 25, or 50 μM) for 72 h in Mueller–Hinton broth containing 10% bovine serum. RNA was extracted and RT-PCR was performed for various genes related to replication (*dna*A, *dna*B, *dna*E, *dna*N, *dna*Q, *pol*A, and *hol*B) and transcription (*rpo*A, *rpo*B, *rpo*D, and *rpo*N). Results showed that mRNA expression levels of *dna*E, *dna*N, *dna*Q, and *hol*B among replication genes of *H. pylori* were decreased in *H. pylori* treated with hesperetin (Figure 2A,C). Furthermore, hesperetin reduced the expression levels of *rpo*A, *rpo*B, *rpo*D, and *rpo*N genes related to the transcription of *H. pylori* (Figure 2B,C). These results suggest that the inhibitory mechanism of *H. pylori* growth by hesperetin is partially suppressed by the downregulation of replication and transcription genes essential for the growth of *H. pylori*.

### 2.2. Downregulation of Urease, Motility, and Adhesion of H. pylori Treated with Hesperetin

**Figure 3:**
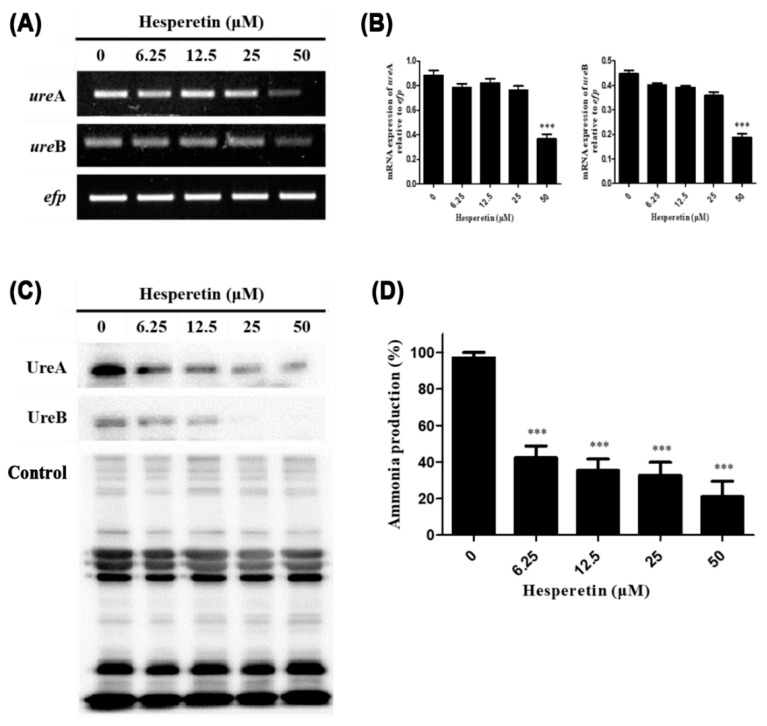
Downregulation of the inhibitory effect of hesperetin on the expression and activity of urease in *H. pylori*. (**A**) *H. pylori* was treated with the indicated concentrations (0, 6.25, 12.5, 25 and 50 μM) of hesperetin. Collected RNA was subjected to RT-PCR to detect mRNA expression levels of urease subunits (*ure*A and *ure*B). The expression of *efp* was used as an internal control. (**B**) Each band intensity was normalized to *efp*. (**C**) Bacterial lysates were subjected to Western blotting to detect UreA and UreB proteins. A rabbit anti-*H. pylori* polyclonal antibody was used as an internal control. (**D**) After 72 h, the supernatant was collected and urease activity was determined by measuring the amount of ammonia. Data are presented as mean ± SEM of triplicate experiments and were analyzed by Student’s *t*-test (*** *p* < 0.001).

Various virulence factors are necessary for *H. pylori* to survive and colonize in the gastric mucosa. The urease of *H. pylori* is essential for its survival in an environment with a strong acidity [40,41]. Therefore, mRNA and protein expression levels of urease α and β subunits of *H. pylori* treated with hesperetin were measured by RT-PCR and Western blot, respectively. Expression levels of both UreA and UreB were decreased in *H. pylori* treated with hesperetin (Figure 3A–C).

To ensure that the effect of decreased expression of UreA and UreB on the urease activity of *H. pylori*, the amount of ammonia produced by urease activity was measured. *H. pylori* was treated with hesperetin at each concentration (0, 6.25, 12.5, 25, and 50 μM) and cultured. After urea was added, the amount of ammonia produced was measured. As a result, it was confirmed that ammonia production of *H. pylori* was remarkably reduced by hesperetin (Figure 3D). Based on this result, the ammonia production of *H. pylori*, which can be seen as a result of urease activity, is thought to be due to the reduction of UreA and UreB expression in *H. pylori* by hesperetin.

**Figure 4:**
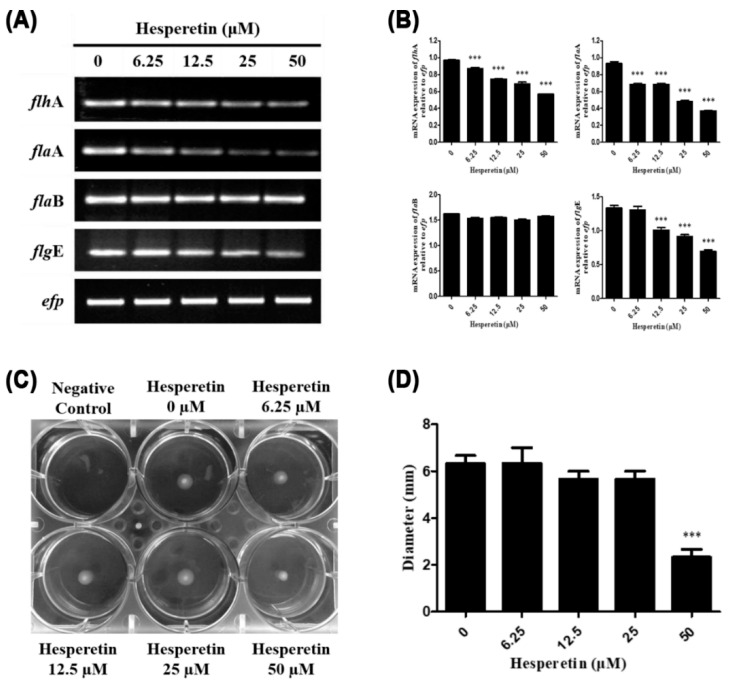
Downregulation of the inhibitory effect of hesperetin on the expression of flagella genes and motility of *H. pylori*. (**A**) *H. pylori* was treated with hesperetin at indicated concentrations (0, 6.25, 12.5, 25, and 50 μM). The collected RNA was subjected to RT-PCR to detect mRNA expression levels of flagella genes (*flh*A, *fla*A, *fla*B, and *flg*E). The expression of *efp* was used as an internal control. (**B**) Each band intensity was normalized to *efp*. (**C**) Mueller–Hinton semi-solid agar containing 0.4% agar and hesperetin at the indicated concentrations (0, 6.25, 12.5, 25, and 50 μM). The negative control was a motility medium not inoculated with *H. pylori*. *H. pylori* was inoculated using a needle and cultured in a CO_2_ incubator at 37 °C for 5 days. After incubation, the radius of *H. pylori* growth spread was measured. (**D**) Data are presented as mean ± SEM of triplicate experiments and were analyzed by Student’s *t*-test (*** *p* < 0.001).

*H. pylori* moves to the gastric epithelium and attaches to epithelial cells. Such attachment is necessary for successful infection. *H. pylori* moves through flagella movements [42]. Therefore, in this experiment, expression levels of genes constituting the flagella (*flh*A, *fla*A, *fla*B, and *flg*E) of *H. pylori* treated with hesperetin were determined by RT-PCR. Expression levels of *flh*A, *fla*A, and *flg*E were decreased by hesperetin treatment (Figure 4A,B). To confirm the effect of the reduction of *flh*A, *fla*A, and *flg*E gene expression on the motility of *H. pylori*, a motility test was performed. The motility medium contained 0.4% agar and hesperetin at different concentrations (0, 6.25, 12.5, 25, and 50 μM). After incubating *H. pylori* for 5 days, it was observed that *H. pylori* grew widely. Its diameter was measured. In this experiment, as the hesperetin concentration increased compared to the control medium, *H. pylori* did not grow widely and its diameter decreased (Figure 4C,D). These results suggest that hesperetin can inhibit the motility of *H. pylori* by reducing the expression of *flh*A, *fla*A, and *flg*E genes in *H. pylori*.

**Figure 5:**
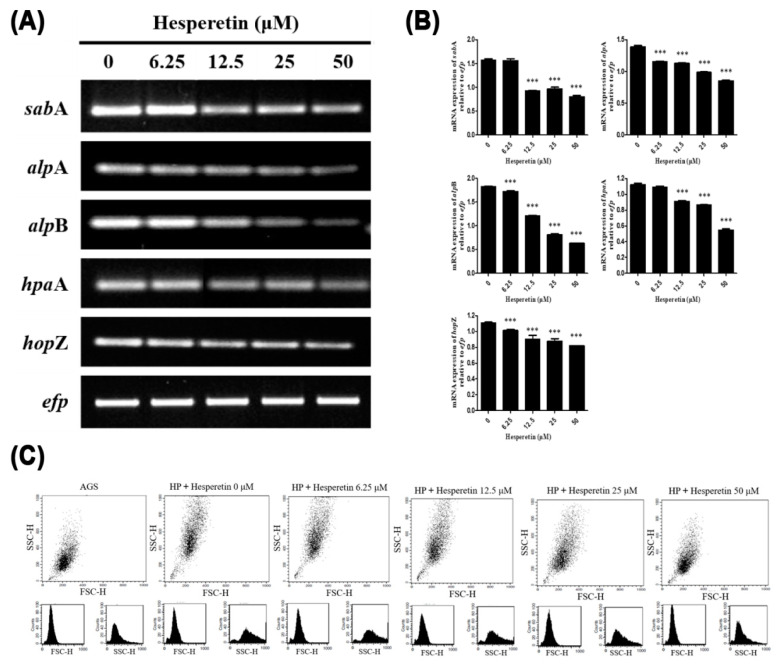
Downregulation of the inhibitory effect of hesperetin on the expression of adherence genes and adhesion of *H. pylori*. (**A**) *H. pylori* was treated with hesperetin at indicated concentrations (0, 6.25, 12.5, 25, and 50 μM). The collected RNA was subjected to RT-PCR to detect mRNA expression levels of adherence genes (*sab*A, *alp*A, *alp*B, *hpa*A, and *hop*Z). The expression of *efp* was used as an internal control. (**B**) Each band intensity was normalized to *efp*. (**C**) *H. pylori* was pretreated with hesperetin at the indicated concentrations (0, 6.25, 12.5, 25, and 50 μM). AGS cells were infected with *H. pylori* (100 MOI) for 30 min in a shaking incubator. After incubation, the cell complexity was analyzed using a flow cytometer. Data are presented as mean ± SEM of triplicate experiments and were analyzed by Student’s *t*-test (*** *p* < 0.001).

The expression of genes related to adhesion closely associated with infection of *H. pylori* was investigated by RT-PCR. Results showed that expression levels of *sab*A, *alp*A, *alp*B, *hpa*A, and *hop*Z genes of *H. pylori* were all decreased by hesperetin treatment (Figure 5A,B). To determine whether the expression of *sab*A, *alp*A, *alp*B, *hpa*A, and *hop*Z genes was involved in cell binding of *H. pylori*, an adhesion assay was performed. *H. pylori* was incubated in a medium including the indicated concentration of hesperetin. *H. pylori* at 100 MOI was then mixed with AGS cells. After reaction for 30 min in a shaking incubator, cell adhesion of *H. pylori* was measured using a flow cytometer. Results showed that AGS cells infected with *H. pylori* had higher side scatter, meaning that the complexity of these cells was higher than that of normal AGS cells because *H. pylori* adhered to AGS cells. For AGS cells infected with *H. pylori* treated with hesperetin, it was observed that as the concentration of hesperetin increased, side scatters were similar to those of normal AGS cells (Figure 5C). This suggested that the reduction of *H. pylori* cell adhesion was due to the inhibition of *sab*A, *alp*A, *alp*B, *hpa*A, and *hop*Z gene expression in *H. pylori* treated with hesperetin. 

In summary, these data indicate that hesperetin may interfere with the survival and colonization of *H. pylori* by reducing the expression and activity of urease, motility, and adhesion of *H. pylori*.

### 2.3. Hesperetin Reduces CagA and VacA Translocation to AGS Cells

CagA and VacA proteins are representative proteins secreted by *H. pylori*. They can destroy gastric epithelial cells and cause gastrointestinal diseases. When CagA protein is injected into the host cell through the type IV secretion system, rearrangement of the actin cytoskeleton will occur, leading to a changed shape of the host cell. Especially, this change of cell morphology induced by CagA is called a hummingbird phenotype [43]. VacA proteins secreted by type V_a_ secretion system can cause the vacuolation and accumulation of many vesicles inside the host cells [44]. Thus, we investigated whether hesperetin could change cell morphology by CagA and VacA proteins and affect the translocation of both proteins into host cells.

**Figure 6:**
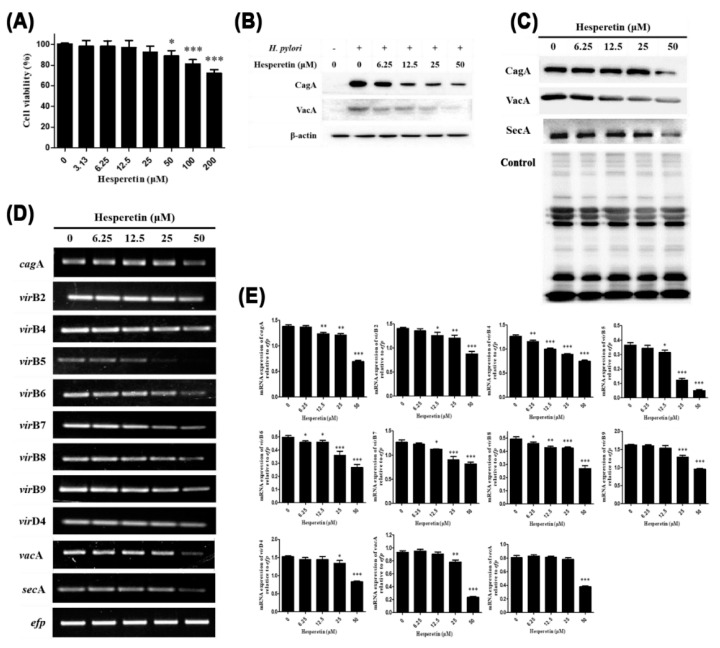
Inhibitory effect of hesperetin on CagA and VacA translocation to the gastric cell line. (**A**) AGS gastric cancer cells were treated with hesperetin at indicated concentrations (0, 3.13, 6.25, 12.5, 25, 50, 100, and 200 μM) for 24 h. Cell viability was then measured by the WST assay. AGS cells were infected with *H. pylori* (100 MOI) and treated with hesperetin at indicated concentrations (0, 6.25, 12.5, 25 and 50 μM) for 24 h. (**B**) After 24 h of incubation, cell lysates were subjected to Western blotting to detect translocated CagA and VacA proteins. β-Actin was used as an internal control. *H. pylori* was treated with hesperetin at the indicated concentrations (0, 6.25, 12.5, 25, and 50 μM) for 72 h. (**C**) Bacterial lysates were subjected to Western blotting to detect CagA, VacA, and SecA. A rabbit anti-*H. pylori* polyclonal antibody was used as an internal control. (**D**) The collected RNA was subjected to RT-PCR to detect mRNA expression levels of *cag*A, T4SS components, *vac*A, and *sec*A genes. The expression of *efp* was used as an internal control. (**E**) Each band intensity was normalized to *efp*. Data are presented as mean ± SEM from three independent experiments and were analyzed by unpaired Student’s *t*-test (* *p* < 0.05, ** *p* < 0.01, and *** *p* < 0.001).

AGS cells were co-cultured with 100 MOI of *H. pylori*, which was exposed to hesperetin at each concentration (0, 6.25, 12.5, 25 and 50 μM) that did not affect cell viability (Figure 6A). After 6 h, the morphological changes of AGS cells induced by *H. pylori* infection were observed under a microscope. Compared to uninfected AGS cells, *H. pylori*-infected AGS cells showed a hummingbird phenotype and vacuolation by CagA and VacA proteins. These morphological changes of AGS cells were relieved by hesperetin treatment in a dose-dependent manner (data not shown). It was assumed that the translocation of CagA and VacA proteins to AGS cells might have decreased by hesperetin treatment. AGS cells infected with *H. pylori* exposed to hesperetin were harvested and Western blot was performed to investigate the amount of translocation of CagA and VacA proteins into AGS cells. Both CagA and VacA proteins were detected in *H. pylori*-infected AGS cells. As expected, the amounts of both proteins were decreased by hesperetin treatment (Figure 6B). In particular, CagA protein was dramatically decreased by hesperetin treatment. These results indicate that hesperetin can reduce the translocation of CagA and VacA proteins into AGS cells and inhibit morphological changes such as hummingbird phenotype and vacuolation caused by *H. pylori* infection.

To discover why the amounts of CagA and VacA proteins were decreased in *H. pylori*-infected AGS cells, the expression levels of CagA and VacA and the secretion system secreting each protein in *H. pylori* treated with hesperetin were measured by RT-PCR and Western blot. Hesperetin reduced mRNA and protein expression levels of CagA and VacA in *H. pylori* (Figure 6C–E). Moreover, mRNA expression levels of the components of T4SS for injecting CagA protein into AGS cells were examined using RT-PCR. Results showed that mRNA expression levels of *virB2*, *vir*B4, *vir*B5, *vir*B6, *vir*B7, *vir*B8, *vir*B9, and *vir*D4 were decreased by hesperetin treatment (Figure 6D,E). Both mRNA and protein expression levels of SecA, a regulator of T5_a_SS in *H. pylori*, were also decreased by hesperetin treatment (Figure 6C–E).

In summary, these data indicated that hesperetin could inhibit the expression of CagA in *H. pylori* and downregulate the T4SS components required for injecting CagA into AGS cells. Hesperetin could also reduce VacA expression and SecA essential for VacA secretion. As hesperetin inhibited the translocation of CagA and VacA proteins, it mitigated the morphological changes of AGS cells induced by *H. pylori* infection.

## 3. Discussion

It is known that *H. pylori* infection is one of the crucial risk factors for gastric cancer. It accounts for 85% of all gastric cancers [45,46]. However, increased prevalence of resistance to antibiotics including clarithromycin used for the treatment of *H. pylori* limits the use of antibiotics as the first-line therapy for *H. pylori* [47]. Therefore, there is a need to develop new therapeutic agents or therapeutic supplements that help eradicate *H. pylori*. Several reports have shown that hesperetin has antibacterial effects on various Gram-positive and Gram-negative bacteria [34,35].

MICs of hesperetin for *H. pylori* reference strains were 50 μM (15.11 μg/mL) and 100 μM (30.23 μg/mL) with the agar dilution method (Figure 1B) and 6.25 μM (1.89 μg/mL) with the liquid dilution method (Figure 1C). In the agar dilution test, the drug was spread to the surface to check bacteria growth. In the broth dilution test, the drug could directly affect the bacteria. Therefore, the MIC with the broth dilution test could be lower than that with the agar dilution test. It has been reported that various flavonoids such as naringenin, quercetin, apigenin, and luteolin could inhibit the growth of *H. pylori* and that the MICs of these substances against *H. pylori* range from 5 to 20 mM [48]. Based on this result, the anti-*H. pylori* activity of hesperetin is more effective than that of the other plant flavonoids reported previously. In addition, the growth of about 20% of *H. pylori* isolated from patients was inhibited by hesperetin at a lower concentration than that for reference strains and about 70% showed the same MICs as the reference strain (Table 2).

During the inhibition of *H. pylori* growth, hesperetin inhibited the expression of the *dna*E, *dna*N, *dna*Q, and *hol*B genes involved in replication, a process essential for *H. pylori* survival (Figure 2A,C). Because *dna*E and *dna*Q are core components of DNA polymerase III, they play an important role in bacterial replication. The *dna*E encodes the α-catalytic subunit of DNA polymerase III holoenzyme. In *Bacillus subtilis*, the polymerase activity of *dna*E is essential for both the initiation and prolongation of DNA replication and the error-prone activity is strongly inhibited by DnaN [56]. Moreover, *dna*N, a DNA polymerase sliding clamp, has been proposed as a target for antibiotics [57,58]. In particular, griselimycin is highly active against *Mycobacterium tuberculosis* by inhibiting DNA polymerase sliding clamp DnaN [59]. The *hol*B gene encodes DNA polymerase III delta subunit [60]. Therefore, the downregulation of the expression of *dna*E, *dna*N, *dna*Q, and *hol*B genes essential for bacterial replication by hesperetin suggests that it might be one of the mechanisms that can inhibit the growth of *H. pylori*.

Furthermore, hesperetin suppressed the expression of *rpo*A, *rpo*B, *rpo*D, and *rpo*N genes related to mRNA synthesis in *H. pylori* (Figure 2B,C). RNA polymerase is involved in bacterial transcription. It is generally composed of α2ββ’ω subunits [9]. RNA polymerase subunit α is encoded by the *rpo*A gene. It is an initiator of RNA polymerase assembly. It serves as a target for transcriptional regulatory proteins [61]. RNA polymerase β is the largest subunit that plays an important role in the assembly of RNA polymerase [62]. Rifampicin is an antibiotic targeting RNA polymerase. It is mainly used to treat *Mycobacterium* infection [63]. Resistance of *M. tuberculosis* to rifampicin is associated with mutations in the *rpo*B gene [64]. *rpo*D and *rpo*N genes encode sigma factors, which are essential regulators of transcription initiation in bacteria that confer promoter recognition specificity on the RNA polymerase core enzyme [65]. These results suggest that downregulation of the RNA polymerase subunit by hesperetin might also be concerned with the inhibition of *H. pylori* growth. Moreover, the diminution of transcription means that various proteins produced by *H. pylori* can also be reduced, consistent with the results of the present study.

Downregulation of the urease subunit by hesperetin may contribute to the inhibition of initial colonization of *H. pylori* in the gastric mucosa and survival in a highly acidic environment (Figure 3). Urease-negative mutant *H. pylori* could not colonize nude mice or gnotobiotic piglets, indicating that urease activity is indispensable for the colonization of *H. pylori* [66,67]. Decreased expression of UreA and UreB by hesperetin reduced the urease activity of *H. pylori* (Figure 3D). Ammonia produced by *H. pylori* urease can directly damage gastric epithelial cells and induce an immune response [68,69]. Moreover, CO_2_ generated by urease is used by *H. pylori* to inhibit peroxynitrite and protect *H. pylori* from oxidative damage, thus increasing the survival rate [70,71]. This means that urease reduced by hesperetin might not only weaken the immune response during *H. pylori* infection, but also potentially interfere with survival in *H. pylori*.

In addition, downregulation of the flagella-related genes *flh*A, *fla*A, and *flg*E by hesperetin might contribute to the inhibition of colonization by inhibiting the migration of *H. pylori* to the gastric epithelium (Figure 4). The *flh*A mutant *Campylobacter jejuni* could not produce flagella. Thus, its colonization is markedly reduced [72]. It has been reported that the *H. pylori* mutant with mutations of the *fla*A and *fla*B genes important for the formation of flagellin constituting flagella lacks motility [13,73]. Although mutant *H. pylori* with mutations of *flg*E, which encodes flagella hook protein, can produce flagella, it lacks motility [74]. These results suggest that downregulation of flagella components by hesperetin might also be involved in the inhibition of *H. pylori* colonization.

Hesperetin inhibited the expression of *sab*A*, alp*A*, alp*B*, hpa*A*,* and *hop*Z genes known as *H. pylori* adherence-related genes. It also interrupted the adhesion of *H. pylori* to gastric epithelial cells (Figure 5). The adhesion ability of *H. pylori* has been proposed as one of the mechanisms of chronic gastritis and gastric cancer caused by *H. pylori* [75]. Chronic infection of *H. pylori* increases inflammation and sialyl-Lewis X expression [17,76]. SabA adhesin mediates *H. pylori* binding to inflamed gastric mucosa by recognizing the sialyl-Lewis a and sialyl-Lewis x antigens [77]. According to Lu et al., alpA and alpB can induce gastric damage by inducing intracellular signaling cascades after *H. pylori* attaches to epithelial cells. Infecting gastric epithelial cells with *alp*A and *alp*B mutant *H. pylori* can lead to decreased IL-6 and IL-8 induction [21]. In wild-type and *hpa*A mutant *H. pylori*-infected mice, the *hpa*A mutant strain could not establish colonization [19]. These results suggest that inhibition of *H. pylori* adhesion by hesperetin can reduce long-term infection and inflammatory response of *H. pylori*, consistent with the results of the present study.

Hesperetin inhibited the translocation of CagA and VacA into host cells (Figure 6B). This might have occurred due to the downregulation of CagA and VacA expression and their secretory system in *H. pylori* (Figure 6C–E). The T4SS of *H. pylori* mediates the injection of CagA into gastric epithelial cells, causing inflammation and gastric cancer [78]. Expression levels of *vir*B2, *vir*B4-9, and *vir*D4 genes were decreased by hesperetin (Figure 6D,E). VirB4, VirB6, VirB8, VirB11, and VirD4 proteins constitute the inner membrane complex of T4SS. In particular, VirB4, VirB11, and VirD4 proteins are inner membrane ATPases required for the assembly of the secretion system, substrate translocation, and pilus formation [79,80]. VirB7, VirB9, and VirB10 compose translocation channels across bacterial membranes. They play an important role in the translocation of CagA [28,29]. The external pilus of T4SS, which directly binds to host cells, is composed of VirB2 and VirB5 proteins. In particular, VirB5 protein is necessary for CagA injection. It induces IL-8 [27,81]. Secretion of the VacA protein is mediated by Sec-dependent T5_a_SS [31]. In this study, expression levels of SecA gene and protein were downregulated by hesperetin treatment (Figure 6C–E). In summary, hesperetin not only inhibited the production of CagA and VacA secreted by *H. pylori*, but also downregulated components of T4SS and T5_a_SS. Thus, the amounts of CagA and VacA proteins translocated into host cells were also reduced.

In this study, inhibitory effects of hesperetin on *H. pylori* growth and *H. pylori*-induced inflammation were ascertained. MICs of hesperetin were validated against *H. pylori* reference strains and clinical isolates. Hesperetin inhibited the growth of *H. pylori* by downregulating the replication and transcription machineries of *H. pylori*. Hesperetin also reduced urease activity by downregulating urease subunit proteins. Hesperetin reduced *H. pylori* motility and adherence activity by downregulating genes constituting flagella and adhesion-related genes required for the successful colonization of *H. pylori*. CagA and VacA as representative virulence factors of *H. pylori* and secretion systems for secreting these proteins were downregulated by hesperetin, which causes decreased translocation of CagA and VacA to host cells.

Further studies using animal infection models are needed to evaluate the stability of hesperetin, as well as the success of using it for *H. pylori* eradication, its anti-inflammatory effect, and its inhibitory effect on the cancer progression caused by long-term *H. pylori* infection.

## 4. Materials and Methods

### 4.1. Bacterial Culture and Collection of H. pylori Clinical Isolates

*H. pylori* reference strains of ATCC 49503, ATCC 43504, ATCC 51932, and ATCC 700392 were purchased from the American Type Culture Collection (ATCC; Manassas, VA, USA). *H. pylori* SS1 strain was obtained from the Korean Type Culture Collection at Gyeongsang National University (Jinju, Korea). *H. pylori* were incubated on Brucella agar plates (BD Biosciences, Braintree, MA, USA) supplemented with 10% bovine serum (BRL Life Technologies, Grand Island, NY, USA) at 37 °C for 72 h under a humidified condition with 5% CO_2_. The number of bacterial particles in the *H. pylori* suspension was set to McFarland 0.33 and cultured in Mueller–Hinton broth supplemented with 10% bovine serum at 37 °C for 72 h under a humidified condition with 5% CO_2_. Gastric biopsy specimens for the isolation of *H. pylori* were collected at Yong-In Severance Hospital, Korea. *H. pylori* clinical strains were isolated from 46 patients undergoing gastroscopic examinations to confirm the infection of *H. pylori*. 

### 4.2. Mammalian Cell Culture

AGS gastric adenocarcinoma cells (ATCC CRL-1739) were cultured in Dulbecco’s modified Eagle’s medium (DMEM; BRL Life Technologies) supplemented with 10% fetal bovine serum (FBS; BRL Life Technologies) and streptomycin–penicillin (100 μg/mL and 100 IU/mL; BRL Life Technologies). AGS cells were incubated at 37 °C in a humidified atmosphere with 5% CO_2_.

### 4.3. Determination of MIC

For the agar dilution test, 10 μL of bacterial suspension was placed on Mueller–Hinton agar supplemented with 10% bovine serum including hesperetin at indicated concentrations (0, 6.25, 12.5, 25, and 50 μM). The final concentration of dimethyl sulfoxide (DMSO) was calculated and treated consistently in all media. Bacteria were incubated for 72 h and MIC was determined based on the lowest concentration showing growth inhibition. For the broth dilution test, various concentrations (0~200 μM) of hesperetin were used for treatment and bacteria were incubated for 72 h. The final DMSO concentration was calculated and treated consistently in all media. The final optical density (600 nm) of the bacterial suspension was measured by spectrophotometry.

### 4.4. Reverse Transcriptase–Polymerase Chain Reaction (RT-PCR)

*H. pylori* ATCC 49503 strain was grown in Mueller–Hinton broth for 72 h with different concentrations (0, 6.25, 12.5, 25, and 50 μM) of hesperetin. Cultured *H. pylori* were washed twice with sterile saline and subjected to total RNA extraction using TRIzol reagent as described in the manufacturer’s instructions. The PCR primer sequences that were used in this study are listed in Table 2 [39,49,50,51,52,53,54,55]. Elongation factor P (*efp*) was used as an internal control. The final PCR products were analyzed using 2% agarose gel electrophoresis, stained with ethidium bromide (EtBr) for 10 min, and destained with distilled water for 20 min. Band intensities of PCR products were analyzed with ImageLab software (Bio-Rad, Hercules, CA, USA).

### 4.5. Western Blotting

Bacteria and AGS cells were washed twice with sterile phosphate-buffered saline (PBS) and lysed with radioimmunoprecipitation assay (RIPA) lysis buffer (Millipore, Billerica, MA, USA) containing a protease inhibitor cocktail (Calbiochem, San Diego, CA, USA). Protein concentrations were determined based on the Lowry method. Antibodies to detect CagA, VacA, and β-actin were purchased from Santa Cruz Biotechnology (Dallas, TX, USA). A polyclonal antibody against the whole *H. pylori* (ATCC 49503) and SecA were produced, as previously described [82]. The polyclonal antibody against whole *H. pylori* was used as an internal control to compare the amount of *H. pylori* protein. β-Actin was used as an internal control for mammalian cell proteins.

### 4.6. Urease Activity Test

*H. pylori* ATCC 49503 strain was grown in Mueller–Hinton broth with different concentrations (0, 6.25, 12.5, 25, and 50 μM) of hesperetin for 72 h. After that, the supernatants were collected. To each sample, 5 μL of 20% urea was added and the samples were incubated at 37 °C for 10 min. Urease activity was confirmed by measuring the amount of ammonia using an Asan Set Ammonia kit (Asan Pharmaceutical, Seoul, Korea) according to the manufacturer’s instructions. Ammonia concentrations in the specimens were calculated using the standard curve.

### 4.7. Motility Test

*H. pylori* ATCC 49503 strain grown in Brucella agar was collected and resuspended in 0.85% sterile saline. The number of bacterial particles in the *H. pylori* suspension was set to McFarland 0.33. *H. pylori* was inoculated using a needle into the motility medium, which was Mueller–Hinton broth supplemented with 10% bovine serum, 0.4% agar, and hesperetin at the indicated concentrations (0, 6.25, 12.5, 25, and 50 μM) in 6-well plates. After 5 days, the diameter *H. pylori* grown on the pate was measured.

### 4.8. Adhesion Activity Test

AGS cells were seeded into DMEM containing 10% FBS without antibiotics for an infection. *H. pylori* cultured in a Mueller–Hinton broth and treated with hesperetin at the indicated concentrations (0, 6.25, 12.5, 25, and 50 μM) was added to AGS cells at 100 MOI and incubated at 37 °C for 30 min in a shaking incubator. After that, AGS cells were washed thrice with PBS and fixed with 1% paraformaldehyde. Cell complexity was then analyzed using a flow cytometer.

### 4.9. Statistical Analysis

Data in bar graphs are presented as mean ± standard error of mean (SEM). All statistical analyses were performed using GraphPad Prism 7.0 software (GraphPad Software, San Diego, CA, USA). All data were analyzed by unpaired Student’s *t*-test, and *p* < 0.05 was considered to be statistically significant (* *p* < 0.05, ** *p* < 0.01, and *** *p* < 0.001). 

## Figures and Tables

**Table 1:** MICs of hesperetin against *H. pylori* clinical isolates.

Hesperetin Concentration (μM)	Number of Strains (%)	
6.25	4	(8.7%)
12.5	3	(6.5%)
25	3	(6.5%)
50	32	(69.6%)
100	4	(8.7%)
Total	46	(100%)

**Table 2:** List of primer sequences and PCR conditions used for RT-PCR.

Primers	Sequences (5′–3′)		Product Length (bp)	Annealing Temperature (°C)	Cycles	Reference
	Forward	Reverse				
DnaA	GGGCATGACTTAGCGGTTA	TTAACGAATTGCACGCCAAC	128	55	27	[49]
DnaB	AATGGGCCGTTTATCGTCTC	CAAATCCGCTTGCAACTACG	231	55	27	
DnaE	AATCCACCGGCTCCAAATAC	GCCAAACAAGTGTGGGAGTA	184	55	27	
DnaN	GTTAGCGGTGGTTGAAAACG	CGGTTTCGCTATGCTCAGAA	233	55	27	
DnaQ	CGCATGAAGCTTTGCAAGAA	GCATAGGCTCTATGGCTGAC	244	55	27	
HolB	TGCAAGCCTTTTTGAACACC	CGCGTTTTGGGCTTCTATAC	196	55	22	
RpoA	AGCGACACGTCTTCAGTAAC	ACAGCACCTTTGATCCCATC	224	55	22	[50]
RpoB	TTTAGGTAAGCGCGTGGATT	AATCAGCTTTGGATGGAACG	301	59	24	
RpoD	TCATCATCATTGCCGACTGG	GTCATGCGCAAACACATTCA	152	55	26	
RpoN	GCCCTTGAAATCGTGCTTAC	ATGATGAGAGCTACCCGACA	250	55	27	
UreA	GCCAATGGTAAATTAGTT	CTCCTTAATTGTTTTTAC	411	40	20	[51]
UreB	TCTATCCCTACCCCACAACC	CCATCCACGAACACATGGTA	252	50	21	
FlhA	TCATTGGAGGGTTTTTAGTGG	GGTGCGAGTGGCGACAAT	155	60	28	[52]
FlaA	TAGACACCACCAACGCTAAA	TGCATTCTAGGGGGTTGTAT	239	62	30	[50]
FlaB	GTCAATGGCGTGAATGATTA	ATTCACGGTCCCAATTTCTA	213	60	30	
FlgE	CCGATCAAATCCTTAACACC	AGGCTTAAAAACATGCGAAC	381	52	30	
SabA	AAAGCATTCAAAACGCCAAC	CCCGCATAAAGACTCCAAAA	163	60	26	[50]
HopZ	GCGCCGTTACTAGCATGATCA	GAAATCTTTCGGCGCGTTT	101	60	26	[53]
HpaA	GAGCGTGGTGGCTTTGTTAGT	TCGCTAGCTGGATGGTAATTCA	90	60	26	
AlpA	GCACGATCGGTAGCCAGACT	ACACATTCCCCGCATTCAAG	90	60	24	
AlpB	ACGCTAAGAAACAGCCCTCAAC	TCATGCGTAACCCCACATCA	82	60	26	
BabA	ATCGATCCACTTCCATCACT	GTTACGCTTTTGCCGTCTAT	292	48	40	
CagA	GTCATAATGGCATAGAACCTGAA	ATTCCCTAGGGCGTCTAAATAA	407	59	21	[39]
VirB2	CAGTCGCCTGACCTCTTTTGA	CGGTCACCAGTCCTGCAAC	156	62	25	
VirB4	GTTATAGGGGCAACCGGAAG	TTGAACGCGTCATTCAAAGC	449	62	37	
VirB5	TACAAGCGTCTGTGAAGCAG	GACCAACCAACAAGTGCTCA	436	62	30	
VirB6	CCTCAACACCGCCTTTGGTA	TAGCCGCTAGCAATCTGGTG	225	62	25	
VirB7	GATTACGCTCATAGGCGATGC	TGGCTGACTTCCTTGCAACA	202	62	25	
VirB8	GTTGATCCTTGCGATCCCTCA	CGCCGCTGTAACGAGTATTG	218	62	25	
VirB9	GCATGTCCTCTAGTCGTTCCA	TATCGTAGATGCGCCTGACC	269	62	25	
VirD4	CCGCAAGTTTCCATAGTGTC	GCGAGTTGGGAAACTGAAGA	263	62	25	
SecA	AAAAATTTGACGCTGTGATCC	CCCCCAAGCTCCTTAATTTC	274	47	27	
VacA	AAACGACAAGAAAGAGATCAGT	CCAGCAAAAGGCCCATCAA	291	57	22	[54]
Efp	GGCAATTTGGATGAGCGAGCTC	CTTCACCTTTTCAAGATACTC	559	59	23	[55]

## Data Availability

The data that support the findings of this study are available from the corresponding author upon reasonable request.
